# Postoperative Joint Line Convergence Angle (JLCA) following high tibial osteotomy is affected not only by preoperative JLCA but also by postoperative hip‐knee‐ankle angle

**DOI:** 10.1002/jeo2.70771

**Published:** 2026-05-25

**Authors:** Dai Sato, Kazunori Yasuda, Eiji Kondo, Jun Onodera, Taku Ebata, Norimasa Iwasaki, Tomonori Yagi

**Affiliations:** ^1^ Department of Orthopaedic Surgery Yagi Orthopaedic Hospital Sapporo Japan; ^2^ Department of Orthopaedic Surgery Hokkaido University Graduate School of Medicine Sapporo Japan; ^3^ Center for Sports Medicine Hokkaido University Hospital Sapporo Japan

**Keywords:** alignment of the lower limb, high tibial osteotomy, hip‐knee‐ankle angle, joint line convergence angle, knee

## Abstract

**Purpose:**

Accurate preoperative planning for high tibial osteotomy (HTO) requires understanding the factors influencing postoperative joint line convergence angle (JLCA). This study aimed to identify independent predictors of postoperative absolute JLCA and its change from preoperative values (ΔJLCA).

**Methods:**

Radiological assessments were performed on 113 knees from 109 patients who underwent inverted V‐shaped high tibial osteotomy (iV‐HTO), both preoperatively and 1 year postoperatively, using radiographs taken in standing and supine positions. Prior to univariate and multiple regression analyses, the knees were divided into two Groups S (preoperative JLCA ≤ 2°) and G (preoperative JLCA > 2°). To explore affecting factors, first, univariate regression analysis was performed to assess the correlation between each of the demographic and radiographic parameters and each of the postoperative JLCA and ΔJLCA. Then, multiple regression analyses were performed separately for postoperative JLCA and ΔJLCA, using the significant parameters identified in the univariate regression analysis as explanatory variables.

**Results:**

In Group G, the postoperative JLCA (mean, 2.5°) was significantly reduced (*p* < 0.0001) compared to the preoperative JLCA (4.4°), while there was no significant difference between the preoperative (1.5°) and postoperative JLCA (1.3°) values in Group S. In Group G, the postoperative JLCA was significantly affected by the preoperative JLCA (*p* < 0.001) and the postoperative HKA (*p* = 0.032). The following multiple regression equation with significant usefulness (*p* < 0.001), the adjusted *R* ^2^ of which was 0.342, was yielded. On the other hand, the postoperative ΔJLCA was significantly affected by the preoperative laxity of the lateral soft tissues (*p* < 0.001) and the preoperative JLCA (*p* = 0.004).

**Conclusion:**

The postoperative absolute JLCA was significantly affected not only by the preoperative JLCA but also by the postoperative HKA. In HTO for OA knees with a large JLCA, maximal JLCA reduction requires a preoperative plan aiming at the greatest clinically acceptable valgus HKA and a suitable surgical procedure.

**Level of Evidence:**

Level II, cohort study.

**Abbreviations**
%MA: percentage of mechanical axis
A‐P: anteroposterior
BMD: bone mineral density
HKA: hip‐knee‐ankle angle
HTO: high tibial osteotomy
iV‐HTO: inverted V‐shaped HTO
JLCA: Joint line convergence angle
KOOS: Knee Injury and Osteoarthritis Outcome Score
LCP: locking compression plate
LLST: laxity of the lateral soft tissues
mLDFA: mechanical lateral distal femoral angle
mMPTA: mechanical medial proximal tibial angle
MOW: medial opening wedge
OA: osteoarthritis
YAM: young adult mean

## INTRODUCTION

**Figure 1:**
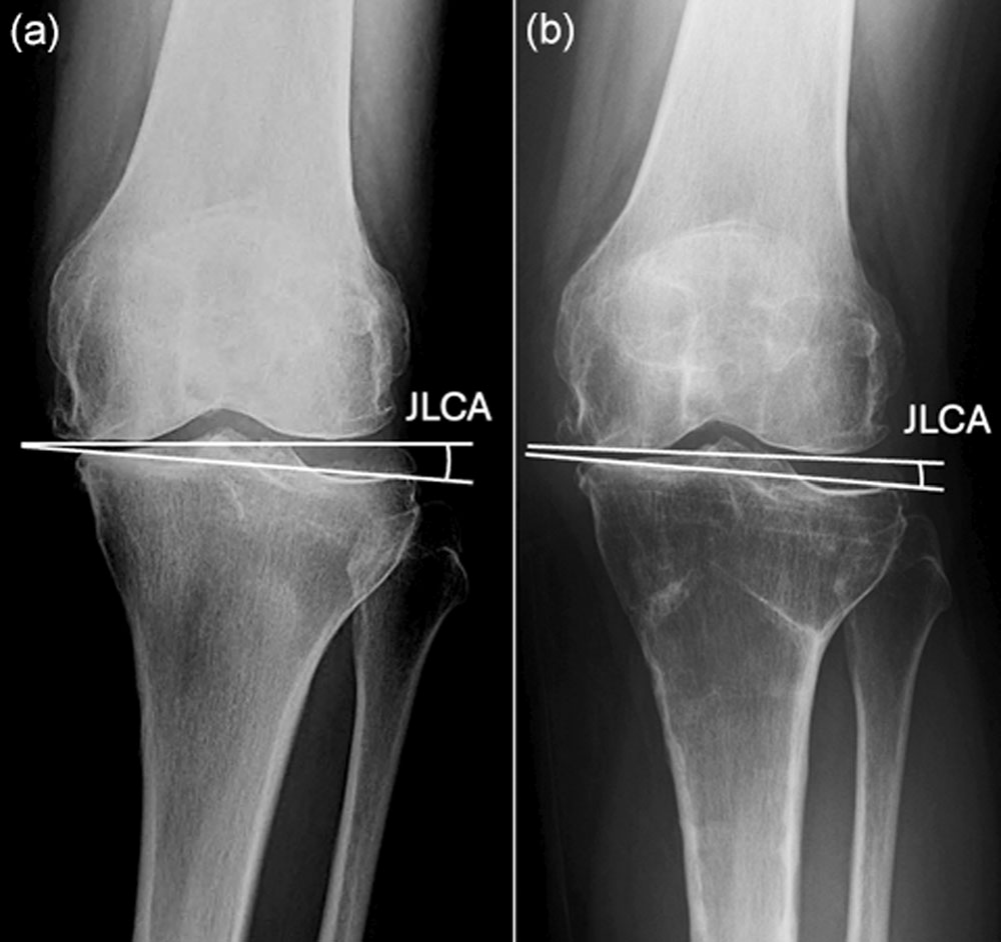
Joint line convergence angle (JLCA) is defined as the angle between the two tangential lines at the distal femoral and proximal tibial articular surfaces. (a) Preoperative, (b) after implant removal following inverted V‐shaped high tibial osteotomy (iVHTO).

High tibial osteotomy (HTO) is an effective surgical treatment for active middle‐aged and elderly patients suffering from osteoarthritis (OA) of the knee with varus deformity. To obtain favourable long‐term clinical outcomes after HTO, it is required to accurately correct the lower limb alignment of a patient, based on appropriate preoperative planning [2, 6, 10]. The varus deformity of the knee with advanced medial OA　is formed by both extra‐articular and intra‐articular deformities [3]. The extra‐articular varus deformity usually occurs in the proximal tibia, and HTO is a surgery to correct this deformity. The intra‐articular deformity, arising from medial joint space narrowing and lateral soft tissue laxity, can be quantified by the joint line convergence angle (JLCA) defined as the angle between two articular tangential lines of the distal femur and proximal tibia (Figure 1) [10, 16, 19, 20, 22].

In preoperative planning for HTO, the tibial correction angle is determined using a full‐length radiograph of the lower extremity, ensuring that the mechanical axis passes through the surgeon's targeted point on the lateral joint surface [19]. However, preoperative planning cannot account for postoperative changes in JLCA, which may occur unpredictably following tibial alignment correction. Subsequently, unexpected changes in JLCA following HTO lead to an overcorrection of the postoperative hip‐knee‐ankle angle (HKA) compared to the planned HKA [12, 15, 23, 26]. To optimize preoperative planning of HTO for knees with substantial preoperative JLCA, a method to predict the postoperative JLCA before surgery is critical; however, no reliable prediction model has been developed yet. Therefore, identifying preoperative and postoperative factors associated with postoperative JLCA is necessary to develop such a model.

Only a few previous studies using medial opening wedge (MOW) HTO investigated factors affecting the postoperative absolute JLCA [4, 8, 22]. For example, Ji et al. [8] and So et al. [23] reported that postoperative JLCA after HTO is primarily associated with preoperative coronal joint‐line parameters, including baseline JLCA and the supine‐to‐standing JLCA discrepancy (reflecting latent soft‐tissue laxity). However, there remains a scarcity of research data on the factors influencing the postoperative JLCA. On the other hand, several studies reported some preoperative factors affecting the postoperative changes in JLCA relative to its preoperative value (abbreviated as ΔJLCA) [4, 8, 12, 16, 17, 22]. Importantly, it remains unclear whether factors associated with postoperative ΔJLCA are the same as those influencing postoperative absolute JLCA, and postoperative variables affecting either outcome have not been definitively identified.

The present study aims to independently identify the factors affecting postoperative absolute JLCA and ΔJLCA through multiple regression analysis. A feature of the present study is the inclusion of patients who underwent inverted V‐shaped HTO (iV‐HTO), a procedure capable of achieving significant correction with fewer associated complications, even in knees with severe varus deformity [14, 29]. Thus, the following hypotheses were formulated in the present study: (1) The postoperative absolute JLCA would be associated with preoperative JLCA and postoperative HKA. (2) The postoperative ΔJLCA would be associated with preoperative JLCA and preoperative lateral soft‐tissue laxity, but not with postoperative alignment‐change parameters. The purpose of this study is to test these hypotheses.

## METHODS

### Study design

A cohort study was conducted using 113 knees of 109 patients who underwent the iV‐HTO fixed with a locking compression plate (LCP) (TriS Inverted‐V Lateral HTO Plate; Olympus Terumo Biomaterials, Tokyo) between April 2021 and March 2023 in our hospital. The following study protocol was approved by the ethics review board of our hospital, and informed consent was obtained from all individual participants.

The indications for the iV‐HTO procedure included (1) a knee with persistent pain due to medial OA that did not improve despite 3 months of nonoperative therapy, (2) a medial OA knee that needed a valgus correction angle >10°, (3) patients who want to return to active daily activities, sports, or labour, and (4) patient age is less than 75 years. The contraindications involved (1) a loss of knee extension <10°, (2) a loss of knee flexion <120°, (3) a knee with a lateral meniscus injury requiring surgical treatment, (4) a knee with severe patellofemoral OA, (5) a knee with cruciate and collateral ligament insufficiency, (6) a history of infection in the knee or the tibia and (7) a history of severe trauma of the leg.

A tibial correction angle was determined for each knee so that the mechanical axis in the corrected limb passed through a point on the lateral tibial plateau, which was 67% lateral to the medial edge of the tibial joint surface, according to the original paper reported by Fujisawa et al. [5]. Two senior orthopaedic surgeons (K.Y. and E.K.) who were sufficiently trained concerning the procedure performed all operations using the previously reported procedure with a LCP [9, 13]. The same rehabilitation protocol was used for all the knees after surgery. Each patient was followed up at the outpatient clinic after discharge.

To measure alignment of the lower limb and geometry of the femur and the tibia in the coronal plane, an anteroposterior (A‐P) digital radiograph of the whole lower limb was taken in standing on both legs immediately before surgery and at the 1‐year follow‐up period. In addition, to evaluate the preoperative laxity of the lateral soft tissues (abbreviated as LLST) around the knee joint in the coronal plane, preoperative A‐P radiographs of the knee were taken in standing and supine positions, and the difference between JLCA values measured on these radiographs was defined as LLST, following the study reported by So et al. [23].

To precisely analyze factors affecting the postoperative JLCA, the knees were divided into two groups: Group S in which a preoperative JLCA was 2° or less and Group G in which a preoperative JLCA was greater than 2°. The boundary value of 2° was chosen because the review study reported by Micicoi et al. [19] proposed that a preoperative JLCA of 2° or less can be considered as normal and that, in this case, lateral soft tissue laxity is negligible in preoperative planning for osteotomy angle. For each group, the authors first examined whether the preoperative JLCA significantly changed following HTO. Then, only in the group that showed significant postoperative changes in JLCA, univariate and multiple regression analyses were performed to investigate the factors affecting the postoperative absolute JLCA. Furthermore, the factors that influenced the postoperative ΔJLCA were explored as well in the same manner.

Finally, the clinical outcomes were evaluated in each patient using Knee Injury and Osteoarthritis Outcome Score (KOOS).

### Surgical procedure for iV‐HTO

**Figure 2:**
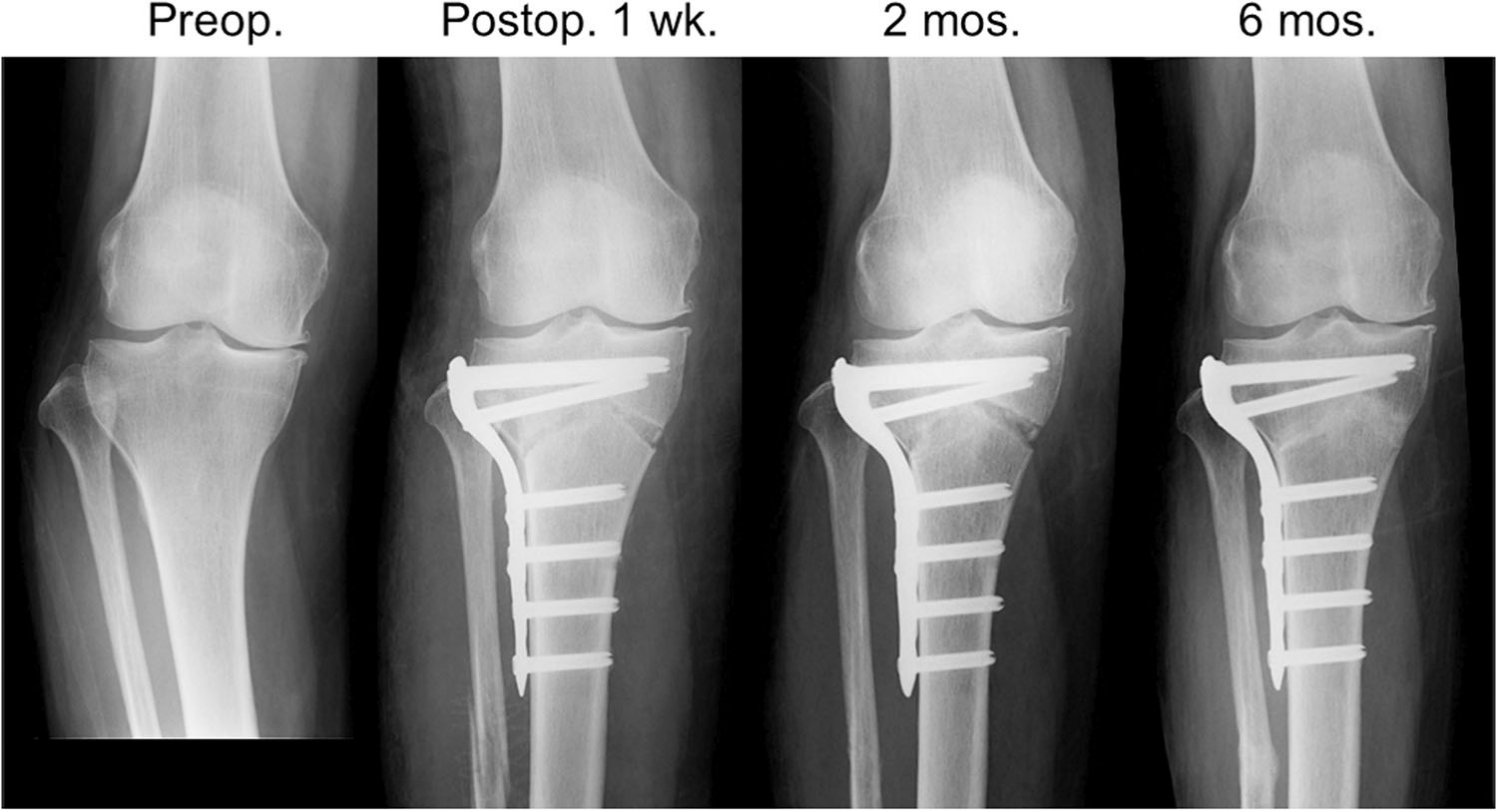
Serial radiographs after inverted V‐shaped high tibial osteotomy (iVHTO). Anteroposterior knee radiographs obtained preoperatively and at 1 week, 2 months, and 6 months postoperatively demonstrate maintenance of correction and progressive healing at the osteotomy site, with complete bone union achieved by 6 months.

The details of the iV‐HTO procedure (Figure 2) were previously reported [9, 13]. First, the fibula was osteotomized at the center of the shaft, using the acute oblique osteotomy and ligation procedure [28, 31]. Then, to perform the tibial osteotomy, a 10‐cm curved skin incision was made on the anterolateral aspect on the proximal tibia. The tibialis anterior muscle was detached from the tibial attachment. Under observation using a C‐arm fluoroscope, a K‐wire was inserted perpendicular to the anterior surface of the proximal tibia and into the apex of the V‐shaped osteotomy. A Protractor‐installed Wire Insertion Guide (Olympus Terumo Biomaterial), in which the angle of the 2 sleeves could be matched to the angle of the planned bone resection, was attached to the apex K‐wire. Using this guide, 2 pairs of K‐wires were inserted from the lateral side of the tibia through these sleeves so that each K‐wire precisely reached the apex wire. The coronal ascending osteotomy parallel to the anterior surface of the tibial tubercle was made using a thin oscillating saw, leaving the proximal part of the tibial tubercle intact with a width of 10 mm. A lateral hemi‐wedge bone resection was made along the 2 pairs of inserted K‐wires, using a thin oscillating saw and a thin chisel. Next, the anteromedial periosteum was longitudinally cut, and the superficial medial collateral ligament were scraped from the tibia. A Parallel Drill Guide (Olympus Terumo Biomaterials) was attached to the apex K‐wire so that the guide was located on the medial osteotomy line. Multiple parallel holes were created in the tibia by inserting a 2 mm–diameter K‐wire into parallel sleeves in this guide. Along these parallel holes, the medial osteotomy was carried out with a thin oscillating saw and a chisel. A valgus correction was made by creating an incomplete fracture at the apex portion of the V‐shaped osteotomy. Temporary fixation was performed using 2 other K‐wires. Fixation of the tibia was achieved using an LCP, which was installed at the lateral side, using the quantitative technique to achieve the correction angle as planned preoperatively [9]. Into an opening space at the medial side of the tibia, morselized bone chips made from the wedged bone removed from the lateral tibia were implanted. The displaced ends of the osteotomized fibula were reduced and securely ligated with a polyester thread, leaving some degree of displacement and angulation [28, 29]. After irrigation, the subcutaneous tissue and the skin were closed.

### Postoperative management

Postoperative rehabilitation was performed according to the previously reported protocols [9, 13]. Quadriceps exercise with quadriceps‐setting and straight leg raising was allowed 1 day after surgery. Passive knee motion from 0 to 90° was allowed during the first 2 weeks, and active knee motion from 0 to 120° was encouraged thereafter. Partial weightbearing using a pair of crutches was allowed at 3 weeks after surgery. Full weightbearing was allowed at 5 weeks after surgery.

### Clinical evaluations for demographic data

Demographic data included gender, age, height, body weight, body mass index, bone mineral density, and OA grade. The bone mineral density (BMD) was measured with DXA Bone Densitometer (Discovery A, Hologic Inc.), and it was shown as the rate (%) to the young adult mean (YAM). On the A‐P radiograph of the knee, the radiological stage of OA was assessed according to the Kellgren‐Lawrence grading system [11].

### Radiological evaluations

**Figure 3:**
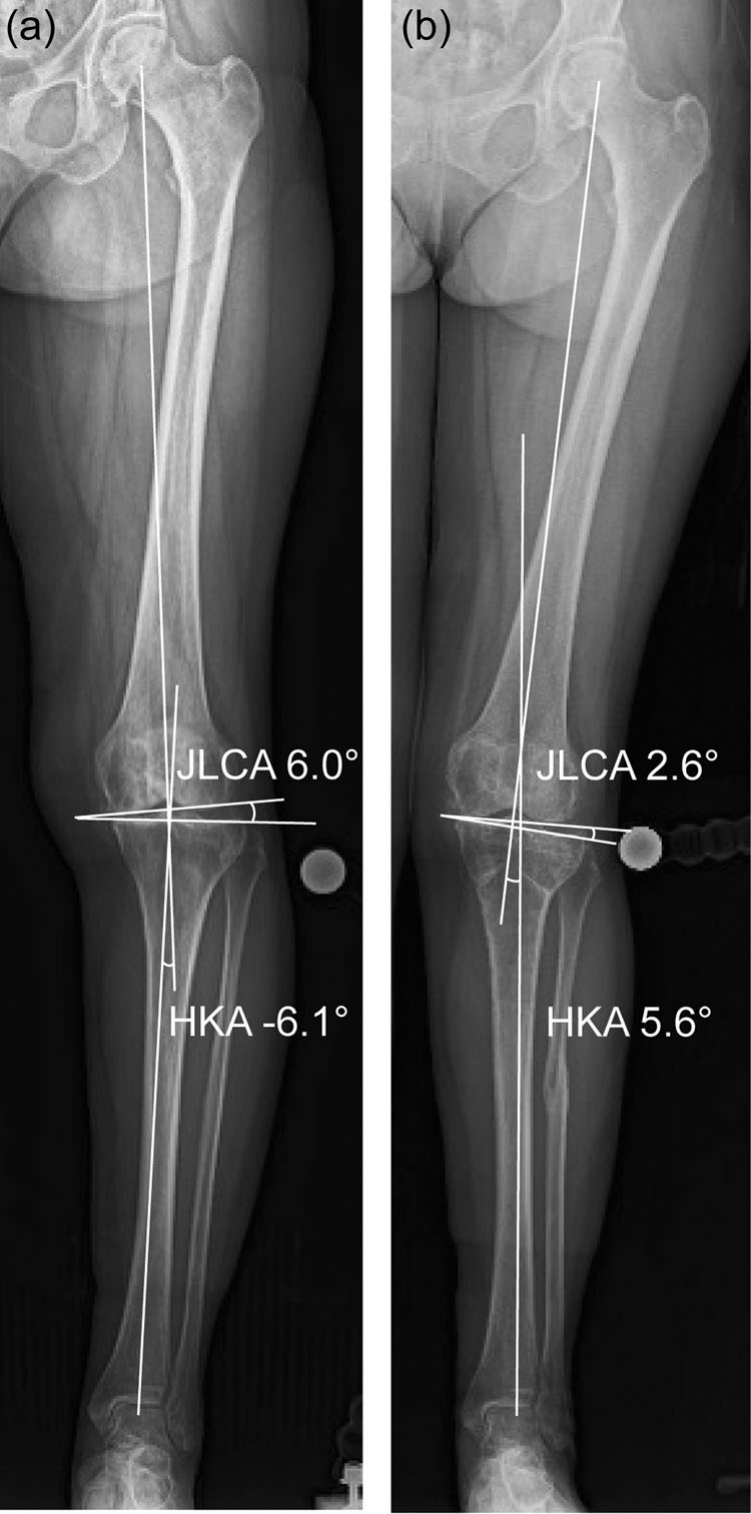
Postoperative changes of JLCA in a representative case (64‐year‐old female) belonging to Group G: (a) Preoperative weight‐bearing radiograph. The measurement of Radiograph A, showing an HKA angle of −6.1° and a JLCA of 6.0°. (b) Postoperative weight‐bearing radiograph taken 1 year after surgery. The measurement of Radiograph B, demonstrating an HKA angle of 5.7° and a JLCA of 2.6°.

On the preoperative and postoperative A‐P digital radiographs (Fujifilm Corporation) of the whole lower limb taken in the standing position, the mechanical lateral distal femoral angle (mLDFA), the mechanical medial proximal tibial angle (mMPTA), the HKA, the percentage of mechanical axis (%MA), and the JLCA were semi‐automatically measured using a commercially available planning software (MediCAD; Hectec) (Figure 3). The lateral opening is designated as the positive value. Concerning the postoperative mLDFA, mMPTA, HKA, and %MA the change relative to the preoperative value was defined as ΔmLDFA, ΔMPTA, ΔHKA, and Δ%MA, respectively. In addition, the preoperative LLST was calculated using the following equation: [23]

LLST = [the JLCA in standing position] – [the JLCA in supine position].

### Statistical analysis

All data were shown as the mean with the standard deviation value. To compare the Groups S and G, the Mann–Whitney *U* test was used for the continuous data, and the Pearson chi‐squared test was used for the discrete data. To examine the influence of the performed HTO to each radiographic parameter, the pre‐ and post‐operative data were compared using the paired *t*‐test.

To explore factors affecting postoperative JLCA and ΔJLCA, univariate regression analysis was performed as the first step, and the correlation between each of the demographic and radiographic parameters and each of the postoperative JLCA and ΔJLCA was assessed. In the second step, multiple regression analyses were performed separately for postoperative JLCA and ΔJLCA, using the significant parameters identified in the univariate regression analysis as explanatory variables. Prior to each analysis, multicollinearity was examined by analyzing correlation coefficients between the significant parameters detected, and if two parameters had a correlation of *r* ≥ 0.7, one of them was excluded [7]. The adjusted coefficient of multiple determination (adjusted *R* ^2^) was used to indicate how much variability in the postoperative JLCA and the postoperative ΔJLCA was accounted for by the selected explanatory variables. To assess statistical significance, standardized regression coefficient (*β*) with 95% confidence intervals and associated *p*‐value were calculated, and a *p*‐value of 0.05 or less was considered statistically significant. A post‐hoc power analysis was performed using G*Power (linear multiple regression, fixed model, *R*² deviation from zero), based on the observed *R*² from the primary regression model, *α* = 0.05, *n* = 49, and two predictors.

A commercially available software programs (JMP Pro 17 for Windows; SAS Institute Japan, and R software 4.4.2; R Foundation for Statistical Computing) were used for statistical calculation. The significance level was set at *p* = 0.05.

## RESULTS

### Demographic data of the patients

All patients were followed up for 1 year after iV‐HTO. Groups S included 64 knees in 63 patients, and Group G involved 49 knees in 46 patients. The preoperative background characteristics of the patients in both groups are shown in Table 1. No significant differences were found in preoperative background characteristics, except for the preoperative OA grade, between Groups S and G. Only the OA grade was significantly more advanced (*p* < 0.001) in Group G than in Group S.

**Table 1:** The preoperative background characteristics of the patients.

Items	Total	Group S	Group G	*p*
Number of knees (patients)	113 (109)	64 (63)	49 (46)	‐‐‐‐‐‐‐
Age (years)	62.3 ± 7.59	62.4 ± 8.42	62.1 ± 6.35	0.779
Male/female (patients)	56/53	33/30	23/23	0.847
Right/left (knees)	57/56	34/30	23/26	0.571
Height (cm)	162.3 ± 9.70	162.6 ± 10.2	162.1 ± 9.19	0.788
Body weight (kg)	69.9 ± 14.1	68.3 ± 13.1	71.1 ± 14.6	0.180
Body mass index (kg/m²)	26.3 ± 3.85	25.6 ± 3.44	26.9 ± 4.09	0.086
Bone mineral density (%)	91.2 ± 14.7	90.8 ± 15.6	91.8 ± 13.5	0.734
OA grade (KL), (knees) Grade 1	5	4	1	<0.001*
Grade 2	45	33	12	
Grade 3	46	24	22	
Grade 4	17	3	14	

*Note*: The *p* values show the results of comparing each item between Groups S and G. Bone mineral density was shown as the rate (%) to the young adult mean (YAM). The continuous data are reported as ‘the mean ± the standard deviation’. *Statistically significant.

Abbreviations: KL, Kellgren‐Lawrence classification; OA, osteoarthritis.

### Preoperative alignment of the lower limb

Preoperatively, the mMPTA values were significantly smaller (*p* = 0.046) in Group G than in Group S. In addition, the preoperative HKA and %MA values showing the degree of varus deformity of the knee was significantly greater (*p* < 0.001) in Groups G than in Group S (Table 2). The preoperative JLCA averaged 1.46° in Group S and 4.41° in Group G with a significant difference between the groups (*p* < 0.001). The preoperative LLST averaged 1.00° in Group S and 2.09° in Group G with a significant difference between the groups (*p* < 0.001).

**Table 2:** Comparisons of the preoperative and postoperative radiographic parameters concerning the alignment of the lower limb between Groups S and G.

Items	Evaluation time	Group S (*n* = 64)	Group G (*n* = 49)	*p* value
mLDFA (°)	Preoperative	87.4 ± 1.8	86.8 ± 1.8	0.062
	Postop. 1Y	87.8 ± 2.1	87.4 ± 1.9	0.374
	*p* value	0.183	0.135	‐‐‐‐‐‐‐‐‐‐‐‐‐‐
mMPTA (°)	Preoperative	84.7 ± 2.2	83.8 ± 1.9	0.046*
	Postop. 1Y	91.7 ± 2.5	93.0 ± 2.5	0.007*
	*p* value	<0.0001*	<0.0001*	‐‐‐‐‐‐‐‐‐‐‐‐‐‐‐
HKA (°)	Preoperative	−4.7 ± 3.3	−6.8 ± 3.2	0.001*
	Postop. 1Y	2.9 ± 2.4	3.5 ± 2.5	0.224
	*p* value	<0.0001*	<0.0001*	‐‐‐‐‐‐‐‐‐‐‐‐‐‐
%MA (%)	Preoperative	28.3 ± 11.6	17.9 ± 14.5	<0.001*
	Postop. 1Y	60.8 ± 9.8	62.7 ± 10.7	0.342
	*p* value	<0.0001*	<0.0001*	‐‐‐‐‐‐‐‐‐‐‐‐‐‐
JLCA (°)	Preoperative	1.5 ± 0.8	4.4 ± 1.4	<0.001*
	Postop. 1Y	1.3 ± 1.1	2.5 ± 1.5	<0.001*
	*p* value	0.154	<0.0001*	‐‐‐‐‐‐‐‐‐‐‐‐‐‐
LLST (°)	Preoperative	1.00 ± 0.99	2.09 ± 1.37	<0.001*

*Note*: The continuous data are reported as ‘the mean ± the standard deviation’. *Statistically significant.

Abbreviations: %MA, percentage of mechanical axis; HKA, hip‐knee‐ankle angle; JLCA, joint line convergence angle; LLST (laxity of lateral soft tissues), the difference in the preoperative JLCA between the supine and standing radiographs; mLDFA, mechanical lateral distal femoral angle; mMPTA, mechanical medial proximal tibial angle.

### Postoperative Changes in the JLCA and other parameters after HTO

In Group G, the postoperative JLCA (2.5 ± 1.5°) was significantly reduced (*p* < 0.0001) compared to the preoperative JLCA (4.4 ± 1.4°), while there was no significant difference between the preoperative and postoperative JLCA values in Group S (Table 2).

The intraoperative correction angle of the tibia in Group G (10.5 ± 2.73°) was significantly greater (*p* = 0.003) that in Group S (8.80 ± 3.03°). Postoperatively, the mMPTA was significantly greater (*p* = 0.007) in Groups G than in Group S (Table 2). There were no significant differences in the post‐operative HKA and %MA values between Groups G and S (Table 2).

### Univariate regression analysis in Group G

Univariate regression analyses were performed to examine the relationship between either postoperative JLCA or ΔJLCA and each of the other preoperative and postoperative measurements individually (Table 3). The postoperative JLCA was significantly correlated with the preoperative JLCA (*R* = 0.547, *p* < 0.001), the postoperative HKA (*R* = −0.309, *p* = 0.032), and the postoperative %MA (*R* = −0.369, *p* = 0.009). On the other hand, the postoperative ΔJLCA was significantly correlated with the preoperative JLCA (*R* = 0.411, *p* = 0.004), the preoperative LLST (*R* = 0.531, *p* = 0.002), the postoperative ΔHKA (*R* = −0.326, *p* = 0.024), and the postoperative Δ%MA (*R* = −0.344, *p* = 0.017).

**Table 3:** Univariate linear regression analyses were performed in Group G with each of the postoperative JLCA and the postoperative ΔJLCA as the objective variable.

	Objective variables			
Explanatory variables	Postoperative JLCA		Postoperative ΔJLCA	
	Coefficient (*R*)	*p* value	Coefficient (*R*)	*p* value
Age	0.077	0.603	−0.182	0.214
Sex, male/female	0.053	0.719	−0.004	0.980
BMI	−0.011	0.941	0.116	0.631
Preoperative				
JLCA	0.547	<0.001*	0.411	0.004*
LLST	0.107	0.562	0.531	0.002*
HKA	−0.218	0.136	−0.228	0.118
%MA	−0.239	0.102	−0.239	0.101
mLDFA	0.100	0.500	0.080	0.588
mMPTA	−0.020	0.893	−0.080	0.588
Intraoperative				
Correction angle	0.048	0.747	0.031	0.836
Postoperative				
HKA	−0.309	0.032*	0.237	0.105
(ΔHKA)	0.048	0.748	−0.326	0.024*
％MA	−0.369	0.009*	0.178	0.227
(Δ％MA)	0.034	0.820	−0.344	0.017*
mLDFA	−0.039	0.794	0.108	0.464
(ΔmLDFA)	0.154	0.296	−0.034	0.819
mMPTA	0.235	0.112	−0.062	0.677
(ΔMPTA)	−0.216	0.144	−0.012	0.936

*Note*: Each row shows the *R* and *p*‐value in the analysis with each item as a potential explanatory variable. *Statistically significant.

Abbreviations: %MA, percentage of mechanical axis; HKA, hip‐knee‐ankle angle; JLCA, joint line convergence angle; LLST, difference in the preoperative JLCA between the supine and standing radiographs; mLDFA, mechanical lateral distal femoral angle; MPTA, medial proximal tibial angle; Resection angle, angle of a lateral bony wedge resected in iV‐HTO.

### Multiple regression analysis in Group G

(1) Factors affecting the postoperative JLCA

Before multiple regression analyses were performed with the postoperative JLCA as the objective variable, the dependency of the potentially significant explanatory variables (preoperative JLCA, postoperative HKA, and postoperative %MA) was tested with the univariate regression analyses. Among them, there was a significant correlation between the postoperative HKA and the postoperative %MA (*R* = 0.97, *p* < 0.0001). Therefore, the multivariate regression analysis was performed with the preoperative JLCA and one of the postoperative HKA and %MA as explanatory variables (Table 4). In the results, the postoperative JLCA (JLCA_postop_) was significantly influenced by the preoperative JLCA (JLCA_preop_, *p* < 0.001) and the postoperative HKA (HKA_postop_, *p* = 0.032). Accordingly, the following multiple regression equation was derived:

$$\left[JLCA_{postop}\right]=0.551\times \left[JLCA_{preop}\right]-0.169\times \left[HKA_{postop}\right]+0.746$$

**Table 4:** Multiple regression analysis with the postoperative JLCA and ΔJLCA as the objective variable.

Objective variable	Explanatory variable	Standardized coefficients (β) (95%CI)	Adjusted R^2^	*p*	VIF
Post JLCA	Pre JLCA	0.551 (0.290 to 0.811)	0.342	<0.001*	1.092
	Post HKA	−0.169 (−0.328 to −0.010)		0.037*	1.092
Post JLCA	Pre JLCA	0.475 (0.175 to 0.775)	0.246	0.002*	1.054
	Post %MA	−0.037 (−0.078 to 0.003)	0.069	1.054	
Post ΔJLCA	Pre JLCA	0.397 (0.133 to 0.660)	0.207	0.003*	1.063
	Post ΔHKA	−0.080 (−0.189 to 0.027)		0.140	1.063
Post ΔJLCA	Pre JLCA	0.390 (0.124 to 0.655)	0.208	0.004*	1.080
	Post Δ%MA	−0.018 (−0.043 to 0.005)		0.133	1.080
Post ΔJLCA	Pre LLST	0.484 (0.224 to 0.744)	0.294	<0.001*	1.121
	Post ΔHKA	−0.067 (−0.169 to 0.0351)		0.192	1.121
Post ΔJLCA	Pre LLST	0.490 (0.219 to 0.761)	0.268	<0.001*	1.114
	Post Δ%MA	−0.014 (−0.038 to 0.009)		0.226	1.114

*Note*: The explanatory variables were determined, based on the univariate regression analyses. The Variance Inflation Factor (VIF) values indicate no multicollinearity between the explanatory variables. *Statistically significant.

Abbreviations: %MA, percent of mechanical axis; CI, confidence interval; HKA, hip‐knee‐ankle angle; JLCA, joint line convergence angle; LLST, laxity of the lateral soft tissues.

The adjusted *R* ^2^ of this equation was 0.342, which was statistically significant (*p* < 0.001). Furthermore, the error in predicting postoperative JLCA values using this regression equation was calculated to be −0.08 ± 1.23°. The achieved power was 0.99 based on the observed model fit (*R*² = 0.342; *f*² = 0.520).

(2) Factors affecting the postoperative ΔJLCA

Multiple regression analyses were performed with the postoperative ΔJLCA as the objective variable. Among the potentially significant explanatory variables, there was a significant correlation between the preoperative JLCA and the preoperative LLST (*R* = 0.531, *p* = 0.002) and between the postoperative ΔHKA and the postoperative Δ%MA (*R* = 0.871, *p* < 0.001). Therefore, the multivariate regression analyses were performed with two explanatory variables: one selected from the preoperative JLCA and LLST, and another selected from the postoperative ΔHKA and Δ%MA (Table 4). The preoperative LLST (*p* < 0.001) and preoperative JLCA (*p* = 0.004) were significant explanatory variables affecting postoperative ΔJLCA, respectively. However, the postoperative ΔHKA or Δ%MA was not a significant explanatory variable.

### Clinical outcomes

Clinical outcomes are summarized in Table 5. In both Groups S (*n* = 64) and G (*n* = 49), all KOOS subscales (Symptoms, Pain, ADL, Sports/Rec, and QOL) improved significantly from preoperative assessment to 1 year postoperatively (all within‐group *p* < 0.001) (Table 5). Several KOOS subscales were lower in Group G than in Group S preoperatively; however, no significant between‐group differences were observed at 1 year postoperatively.

**Table 5:** Pre‐ and postoperative KOOS subscale scores in Groups S and G.

Items	Evaluation time	Group S (*n* = 64)	Group G (*n* = 49)	*p* value
KOOS Symptom	Preoperative	66.7 ± 18.8	57.2 ± 18.7	0.009
	Postop. 1Y	85.5 ± 11.1	83.3 ± 10.7	0.424
	*p* value	<0.001*	<0.001*	‐‐‐‐‐‐‐‐‐‐‐‐‐‐‐
KOOS Pain	Preoperative	58.6 ± 19.8	54.3 ± 19.6	0.251
	Postop. 1Y	87.7 ± 10.6	84.7 ± 12.0	0.300
	*p* value	<0.001*	<0.001*	‐‐‐‐‐‐‐‐‐‐‐‐‐‐
KOOS, ADL	Preoperative	72.8 ± 16.8	64.7 ± 17.1	0.013
	Postop. 1Y	90.8 ± 8.8	86.5 ± 9.3	0.058
	*p* value	<0.001*	<0.001*	‐‐‐‐‐‐‐‐‐‐‐‐‐‐
KOOS, Sports/Rec	Preoperative	38.1 ± 24.3	27.7 ± 22.1	0.022
	Postop. 1Y	65.4 ± 21.9	54.8 ± 27.1	0.102
	*p* value	<0.001*	<0.001*	‐‐‐‐‐‐‐‐‐‐‐‐‐‐
KOOS, QOL	Preoperative	38.6 ± 21.1	26.4 ± 16.0	0.001
	Postop. 1Y	68.5 ± 22.3	68.3 ± 18.3	0.972
	*p* value	<0.001*	<0.001*	

*Note*: Continuous variables are presented as mean ± standard deviation. Between‐group *p* values compare Groups S and G at each time point. Within‐group *p* values compare preoperative and 1‐year postoperative scores within each group. *Statistically significant.

Abbreviations: ADL, activities of daily living; KOOS, Knee Injury and Osteoarthritis Outcome Score; QOL, quality of life.

### Relationship between the postoperative JLCA and clinical outcomes

Univariate regression analyses were performed to examine the relationship between either postoperative JLCA or HKA and each of the postoperative KOOS sub‐scales (Table 6). The postoperative JLCA was significantly correlated with the pain subscale (*R* = −0.292, *p* = 0.041). The postoperative HKA was significantly correlated with the pain subscale (*R* = 0.321, *p* = 0.024) and symptoms and stiffness subscale (*R* = 0.329, *p* = 0.020).

**Table 6:** Univariate linear regression analyses were performed in Group G using each KOOS subscale and postoperative JLCA or HKA as the objective variable.

	Objective variables			
Explanatory variables	Postoperative JLCA		Postoperative HKA	
	Coefficient (*R*)	*p* value	Coefficient (*R*)	*p* value
KOOS Pain	−0.292	0.041*	0.321	0.024*
KOOS symptoms and stiffness	−0.206	0.155	0.329	0.020*
KOOS ADL	−0.202	0.163	0.222	0.123
KOOS Sports and recreation	−0.244	0.091	0.142	0.327
KOOS QOL	0.029	0.843	−0.061	0.677

*Note*: Each row presents the correlation coefficient (*R*) and *p*‐value for the analysis, with each item considered as a potential explanatory variable. *Statistically significant.

Abbreviation: KOOS, Knee Injury and Osteoarthritis Outcome Score.

Although postoperative JLCA showed a statistically significant but weak correlation with KOOS Pain (*r* = −0.292), we further examined whether postoperative JLCA was independently associated with KOOS Pain. In a multivariable linear regression model including postoperative JLCA, Kellgren–Lawrence grade, BMI, age, and postoperative HKA, postoperative JLCA was not significantly associated with KOOS Pain (*β* = −1.133, 95% CI − 3.721 to 1.456; *p* = 0.382) (Table 7). No covariate reached statistical significance in this model.

**Table 7:** Multivariable linear regression analysis for KOOS Pain at 1 year postoperatively.

Explanatory variable	Estimate	95% CI	*p* value
Postoperative JLCA	−1.133	−3.721 to 1.456	0.382
K‐L grade	−2.732	−7.328 to 1.864	0.237
BMI	−0.373	−1.268 to 0.521	0.405
Age	0.006	−0.570 to 0.584	0.980
HKA	1.076	−0.462 to 2.615	0.165

*Note*: The dependent variable was KOOS Pain at 1 year. Independent variables included postoperative JLCA, K‐L grade, BMI, age, and postoperative HKA. *β* indicates the unstandardized regression coefficient.

Abbreviations: BMI, body mass index; HKA, Hip‐Knee‐Ankle angle; JLCA, Joint Line Convergence Angle; K‐L, Kellgren–Lawrence; KOOS, Knee Injury and Osteoarthritis Outcome Score.

## DISCUSSION

The most important findings in the present study are as follows: First, the postoperative JLCA in Group G (with preoperative JLCA > 2°) was significantly reduced following the HTO surgery, whereas the JLCA in Group S (preoperative JLCA < 2°) was not significantly altered postoperatively. These results suggest that exploring factors affecting postoperative JLCA following HTO in Group S would not be rational. Secondly, the univariate and multiple regression analyses were performed in Group G. As a result, the postoperative JLCA was significantly influenced not only by the preoperative JLCA but also by the postoperative HKA. A statistically significant multiple regression equation was subsequently derived using these 2 factors as explanatory variables. Thirdly, the postoperative ΔJLCA was significantly affected by the preoperative LLST and the preoperative JLCA, respectively. However, the parameters concerning the postoperative alignment, including the postoperative ΔHKA or Δ%MA, were not significant explanatory variables affecting postoperative ΔJLCA. These findings offer new knowledge to deeply understand the preoperative and postoperative factors affecting postoperative JLCA and ΔJLCA following HTO.

An important feature of this study design is that the knees were divided into two groups: S (preoperative JLCA < 2°) and G (preoperative JLCA > 2°) prior to univariate and multiple regression analyses. Postoperative JLCA did not significantly change after HTO in Group S. This result indicates that it does not make rational sense to explore factors affecting the postoperative JLCA following HTO in Group S. This result is consistent with those reported by Micicoi et al. [19], who described that, when a preoperative JLCA was 2° or less, lateral soft tissue laxity was negligible in preoperative planning for osteotomy angle. On the other hand, postoperative JLCA significantly changed after HTO in Group G. Therefore, in the present study, the investigation for factors affecting postoperative JLCA was performed only in Group G. The authors believe that this is a logically appropriate analysis design to obtain correct statistical results. In previously reported studies [1, 12, 16, 17, 18, 20, 27], factors affecting postoperative JLCA and ΔJLCA were statistically explored in all knees that underwent HTO, including those corresponding to Group S in the present study. Such study designs may have reduced the precision of regression analyses in identifying factors affecting postoperative JLCA or ΔJLCA. Accordingly, the predictors identified in this study—and the resulting regression equation—should be interpreted as applicable only to patients with preoperative JLCA > 2° (Group G) and should not be directly generalized to all HTO patients, particularly those with preoperative JLCA ≤ 2° (Group S).

The present study demonstrated that, in Group G, the postoperative JLCA following HTO is significantly influenced not only by the preoperative JLCA but also by the postoperative HKA. The preoperative JLCA has been emphasized as an important factor affecting postoperative JLCA and ΔJLCA in previous studies [4, 8, 12, 16, 17, 20, 23, 25]. However, the influence of postoperative HKA on postoperative JLCA has not been clearly demonstrated. Our findings suggest that postoperative JLCA can be modulated through the achieved degree of valgus correction, as reflected in the postoperative HKA. This finding indicates that HTO surgery can affect postoperative JLCA through the degree of lower limb alignment correction. In the present study, a statistically significant multivariable regression equation was derived using preoperative JLCA and postoperative HKA as explanatory variables. Importantly, we also incorporated a surrogate measure of coronal‐plane laxity of the lateral soft tissues using LLST, defined as LLST = (JLCA in standing) − (JLCA in supine). Nevertheless, the model's explanatory power was limited (adjusted R² = 0.342), indicating substantial unexplained variance and suggesting additional determinants such as sagittal‐plane or rotational instability and other anatomic parameters (e.g., posterior tibial slope or rotational alignment). Further studies are needed to validate the clinical utility of this regression equation.

In the present study, it was noted that change in HKA (ΔHKA) was not a significant factor affecting postoperative JLCA. This finding suggests that, in HTO for medial OA knees with severe varus deformity, the postoperative JLCA is determined by the degree of valgus alignment ultimately achieved by HTO, not by the amount of change in valgus alignment made by HTO. This means that, in HTO for patients with a large JLCA, sufficient JLCA reduction cannot be achieved unless adequate postoperative valgus HKA correction is obtained, even if the postoperative ΔHKA is relatively large. Therefore, when planning HTO surgery for medial OA knees with severe varus deformity, it is crucial to select a procedure that fully achieves the preoperatively planned correction without complications. Then, the present study showed that the iV‐HTO procedure successfully corrected the preoperative severe varus deformity (mean, 6.8° varus) into adequate valgus alignment (mean, 3.5° valgus) after surgery without few complications. Recent clinical studies have reported that few complications were observed following the major correction of the tibia using this iV‐HTO procedure [14, 28, 31]. Therefore, the authors recommend the iV‐HTO procedure as one of the surgical options for medial OA knees with severe varus deformity.

The present study indicated that the postoperative ΔJLCA was significantly affected by the preoperative LLST and the preoperative JLCA, respectively. Then, it should be noted that the preoperative LLST was significantly correlated with the preoperative JLCA. In previous studies, So et al. [23] reported that the preoperative LLST played a significant role as a predictor of the postoperative ΔJLCA. Lee et al. [17] emphasized that the preoperative ‘latent medial laxity’ significantly affected the postoperative ΔJLCA. Na et al. [20] stated that the postoperative ΔJLCA was significantly affected by the preoperative JLCA values measured on radiographs taken in supine and standing positions. The results of the present study were largely consistent with those of previous studies. On the other hand, impact of the postoperative alignment on the postoperative ΔJLCA was not pointed out in the previous studies [17, 20, 23]. The present study clearly showed that the postoperative ΔHKA was not a significant factor affecting the postoperative ΔJLCA in the multiple regression analysis, although it was suggested as one of the potential factors in the univariate regression analysis. Although Ji et al. [8] reported an association between Δ%MA and ΔJLCA, discrepancies across studies may reflect differences in measurement protocols and the small absolute magnitude of ΔJLCA, which makes results sensitive to minor measurement error.

In the present study, the significant factors affecting the postoperative absolute JLCA and the postoperative ΔJLCA were not identical. For example, the postoperative HKA was a significant factor for the postoperative JLCA, but not for the postoperative ΔJLCA. Furthermore, the postoperative ΔHKA was a significant factor for the postoperative ΔJLCA, but not for the postoperative absolute JLCA. This finding suggests that postoperative JLCA cannot be reliably predicted based solely on the knowledge of postoperative ΔJLCA, which is reported in several previous studies. Thus, the authors believe that the findings of this study will contribute to a deeper understanding of the factors influencing both postoperative JLCA and postoperative ΔJLCA following HTO.

Clinical relevance of this study is as follows: First, although postoperative JLCA showed a statistically significant but weak correlation with KOOS Pain (*r* = −0.292), it was not independently associated with KOOS Pain after adjustment for KL grade, age, BMI, and postoperative HKA (*β* = −1.133, 95% CI − 3.721 to 1.456; *p* = 0.382), indicating that the bivariate association likely reflects confounding/shared variance. Accordingly, postoperative JLCA should be interpreted as an adjunctive parameter reflecting coronal‐plane laxity and the correction strategy rather than a stand‐alone determinant of pain outcomes, particularly in patients with a large preoperative JLCA. Previous studies have reported that the postoperative HKA is the most significant factor affecting the long‐term outcome of LCWHTO [5, 21, 24, 30]. Therefore, achieving an appropriate postoperative HKA while considering JLCA behaviour may help optimize correction and avoid excessive valgus alignment [12, 15, 23, 26]. Second, the present study demonstrated that both the preoperative JLCA and the postoperative HKA were significant factors affecting the postoperative absolute JLCA. Of these two factors, only the postoperative HKA is under the surgeon's control. This fact suggests that, to achieve maximal reduction of JLCA in HTO for OA knees with a large JLCA, the surgeon must not only establish a preoperative plan aiming at the greatest clinically acceptable degree of valgus HKA but also select an appropriate surgical technique to safely execute this plan. However, the greatest clinically acceptable degree of valgus HKA remains unknown. Previous studies on lateral closing wedge HTO have suggested that a valgus correction of 3 to 5° in the HKA is essential for favourable long‐term outcomes [29, 30, 31]. However, it remains unclear whether this valgus range represents the greatest clinically acceptable degree of valgus HKA.

The present study has several limitations. First, it was a retrospective observational study. Second, the number of patients is not sufficient. Although the post‐hoc power analysis suggested adequate power to detect the observed overall model effect, the relatively small sample size of Group G (*n* = 49) may still affect model stability and generalizability. Therefore, the results should be interpreted cautiously and warrant confirmation in larger cohorts. In addition, the explanatory power of the primary regression model was limited (adjusted *R*² = 0.342), leaving substantial unexplained variance. Although coronal‐plane lateral soft‐tissue laxity was partially assessed using LLST (standing−supine JLCA), we did not directly quantify sagittal‐plane anterior–posterior laxity or rotational instability, nor did we evaluate posterior tibial slope or rotational alignment. These unmeasured factors may partly account for the unexplained variance in postoperative JLCA. Thirdly, the one‐year follow‐up period was too short to evaluate long‐term changes in JLCA, although it was adequate for achieving the objectives of the present study. Fourthly, the clinical utility of the regression equation, which was developed to predict postoperative JLCA in the present study, has not yet been validated. Fifthly, this study was unable to clarify why the significant factors influencing postoperative absolute JLCA and ΔJLCA differed. Sixth, this study evaluated only patients treated with iV‐HTO; therefore, the findings may not be generalizable to other HTO techniques (e.g., MOWHTO or LCWHTO). Because many prior JLCA studies have also been technique‐specific, external validation across techniques with comparable correction magnitude and standardized protocols is warranted. Despite these limitations, the authors believe that this study offers new insights into the preoperative and postoperative factors affecting postoperative absolute JLCA and ΔJLCA following HTO.

In conclusion, in patients with preoperative JLCA > 2°, the postoperative absolute JLCA was significantly affected not only by the preoperative JLCA but also by the postoperative HKA. A statistically significant multiple regression equation was subsequently derived using these 2 factors as explanatory variables. Therefore, for HTO surgery in OA knees with a large JLCA (JLCA > 2°), achieving maximal reduction of the JLCA requires both a preoperative plan targeting the greatest clinically acceptable degree of valgus HKA and the selection of an appropriate surgical technique to safely execute this plan. These conclusions apply to the JLCA > 2° subgroup and should not be generalized to all HTO patients.

## AUTHOR CONTRIBUTIONS

Dai Sato collected the data, performed the analysis, and draughted the manuscript. Kazunori Yasuda conceived the research, performed the surgery, and critically revised the manuscript. Eiji Kondo supervised the data analysis. Jun Onodera and Taku Ebata assisted with data collection. Norimasa Iwasaki and Tomonori Yagi contributed to data interpretation.

## FUNDING INFORMATION

The authors have no funding to report.

## CONFLICT OF INTEREST STATEMENT

The authors declare no conflicts of interest.

## ETHICS STATEMENT

Ethical approval for this study was obtained from the institutional review board of Yagi Orthopaedic Hospital (approval No. H29‐0001). The requirement for informed consent was waived by the institutional review board because this study used anonymized retrospective data.

## Data Availability

The datasets that support the findings of this study are available on request from the corresponding author. The data are not publicly available due to patient privacy regulations and institutional ethical restrictions.
